# FORENSIC: an Online Platform for Fecal Source Identification

**DOI:** 10.1128/mSystems.00869-19

**Published:** 2020-03-17

**Authors:** Adélaïde Roguet, Özcan C. Esen, A. Murat Eren, Ryan J. Newton, Sandra L. McLellan

**Affiliations:** a School of Freshwater Sciences, University of Wisconsin–Milwaukee, Milwaukee, Wisconsin, USA; b Department of Medicine, University of Chicago, Chicago, Illinois, USA; University of California San Diego

**Keywords:** microbial source tracking, 16S rRNA gene, high-throughput sequencing, *Bacteroidales*, *Clostridiales*, random forest classification, toolkit

## Abstract

Sewage overflows, agricultural runoff, and stormwater discharges introduce fecal pollution into surface waters. Distinguishing these sources is critical for evaluating water quality and formulating remediation strategies. With the falling costs of sequencing, microbial community-based water quality assessment tools are under development. However, their application is limited by the need to build reference libraries, which requires extensive sampling of sources and bioinformatic expertise. Here, we introduce FORest Enteric Source IdentifiCation (FORENSIC; https://forensic.sfs.uwm.edu/), an online, library-independent source tracking platform based on random forest classification and 16S rRNA gene amplicon sequences to identify in environmental samples common fecal contamination sources, including humans, domestic pets, and agricultural animals. FORENSIC relies on a broad reference signature database of Bacteroidales and Clostridiales, two predominant bacterial groups that have coevolved with their hosts. As a result, these groups demonstrate cohesive and reliable assemblage patterns within mammalian species or among species sharing the same diet/physiology. We created a scalable and extensible platform that we tested for global applicability using samples collected in distant geographic locations. This Web application offers a fast and intuitive approach for fecal source identification, particularly in sewage-contaminated waters.

**IMPORTANCE** FORENSIC is an online platform to identify sources of fecal pollution without the need to create reference libraries. FORENSIC is based on the ability of random forest classification to extract cohesive source microbial signatures to create classifiers despite individual variability and to detect the signatures in environmental samples. We primarily focused on defining sewage signals, which are associated with a high human health risk in polluted waters. To test for fecal contamination sources, the platform only requires paired-end reads targeting the V4 or V6 regions of the 16S rRNA gene. We demonstrated that we could use V4V5 reads trimmed to the V4 positions to generate the reference signature. The systematic workflow we describe to create and validate the signatures could be applied to many disciplines. With the increasing gap between advancing technology and practical applications, this platform makes sequence-based water quality assessments accessible to the public health and water resource communities.

## Abstract (precis)

FORENSIC is an online platform to identify sources of fecal pollution without the need to create reference libraries. FORENSIC is based on the ability of random forest classification to extract cohesive source microbial signatures to create classifiers despite individual variability and to detect the signatures in environmental samples. We primarily focused on defining sewage signals, which are associated with a high human health risk in polluted waters. To test for fecal contamination sources, the platform only requires paired-end reads targeting the V4 or V6 regions of the 16S rRNA gene. We demonstrated that we could use V4V5 reads trimmed to the V4 positions to generate the reference signature. The systematic workflow we describe to create and validate the signatures could be applied to many disciplines. With the increasing gap between advancing technology and practical applications, this platform makes sequence-based water quality assessments accessible to the public health and water resource communities.

## INTRODUCTION

Environmental fecal pollution is recognized worldwide as a major threat to human health that causes millions of deaths in developing countries (1). While the waterborne disease burden is far less in developed countries, chronic fecal pollution is regularly reported (2) and leads to waterborne illness, diminished ecosystem services, and economic loss (3). In urbanized watersheds, fecal contamination can originate from multiple sources, including failing sewage infrastructure and contaminated runoff containing waste from upstream livestock, pets, and wildlife. However, gastrointestinal illness risks may differ according to the pollution source. For example, human exposure to waters contaminated by sewage or cattle waste is estimated to present a greater health risk than similar exposure to avian waste (4, 5). Water quality measures based on traditional fecal indicator bacteria do not identify pollution sources (6). As a result, more advanced methods, including quantitative PCR, have been developed to distinguish fecal contamination sources by targeting marker sequences that are indicative of a specific pollution source (7).

The ever-decreasing cost of sequencing and the development of advanced computational tools has made high-throughput sequencing one of the most efficient ways to study bacterial communities, and it shows promise for microbial source tracking applications (8, 9). Recent studies have used 16S rRNA gene amplicons to successfully characterize human and animal fecal sources in surface waters (10–16). These studies rely on the detection of fecal community signatures shaped by coevolutionary dynamics between hosts and their gut microbiota (17–20). These approaches, including use of the state-of-the-art Bayesian classifier SourceTracker (21), require the creation of a reference sequence library for fecal samples, which entails sampling and sequencing both of source samples and of the water samples of interest. Such additional effort is often beyond the capabilities or resources available to a single investigator. This limitation is compounded when fecal references are geographically distant from samples tested, which can alter the accuracy of source identification (14). To date, a publicly available database that encompasses most common animal sources for fecal contamination does not exist.

Our previous work reported a source tracking method using random forest classification, which offered a rapid, sensitive, and accurate solution for identifying host-microbial signatures in environmental samples (15). In this study, we demonstrated that the two most common gut-associated bacterial orders in mammals, i.e., *Bacteroidales* and *Clostridiales* (18, 22), provide enough host-specific signal to perform accurate source identification. Similar observations were reported at the genus level (23–25), highlighting the fractal nature of specialization between gut microbiota and their hosts. Targeting taxonomically defined groups, rather than studying the whole bacterial community, may reduce the influence of large cross-phylum shifts due to diet or sequencing primer bias in the bacterial community (25) while providing relevant host-associated profiles.

Here, we introduce the Web application FORest Enteric Source IdentifiCation (FORENSIC), an online platform with a user-friendly interface that employs random forest to identify common sources responsible for fecal contamination. FORENSIC gives access to an extensible list of human and animal reference signatures, eliminating the need for users to create their own fecal reference databases. This platform responds to an emerging demand for sequence data in the advancement of water quality testing.

## RESULTS

Fecal gut microbiota were explored in 132 human (sewage) and 395 animal fecal source samples collected around the world, which included but not were not limited to those from cats, cattle, dogs, deer, pigs, horses, goats, sheep, rabbits, raccoons, chicken, and geese. Amplicon sequences generated from the V4, V4V5, and V6 regions of the 16S rRNA gene were used to create and validate global FORENSIC source signatures.

### Host community patterns and performance of global classifiers.

**FIG 1:**
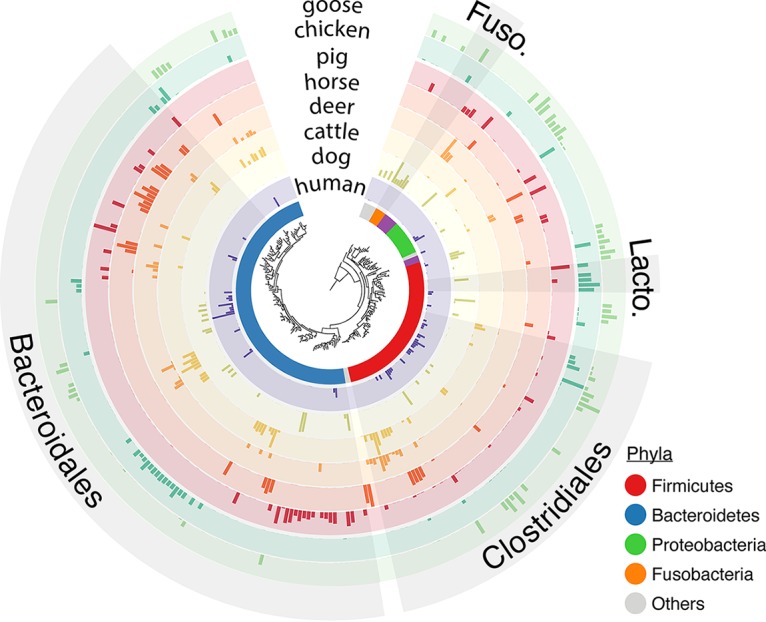
Phylogeny and distribution of the dominant V4 sequences across the fecal microbiota of eight host species. Ten fecal samples were averaged per host, except for dog, chicken, and goose, represented by 7, 6, and 8 samples, respectively. Only sequences (*n* = 287) with an average relative abundance per host of 0.5% are displayed. Colors in the inner circle depict phyla. Gray clades symbolize the dominant bacterial orders for at least one host. Log-transformed relative abundances were normalized by the maximum abundance for each host. Fuso., Fusobacteriales; Lacto., Lactobacillales. The tree was rooted using Halobellus ramosii strain S2FP14 (GenBank accession no. NR_145608.1). Sequences were aligned using MUSCLE implemented in MEGA (61). The tree was generated using the interactive Tree Of Life (iTOL) (62).

**FIG 2:**
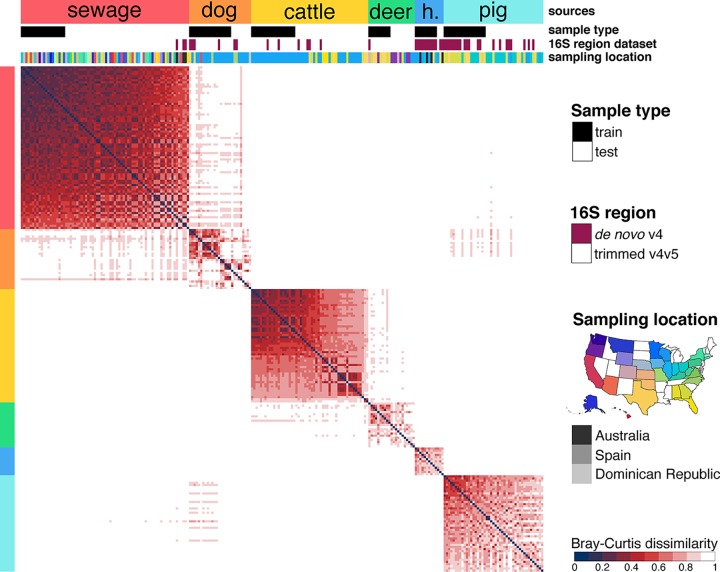
Bray-Curtis analysis of the *Bacteroidales* assemblage for the V4 region of the 16S rRNA gene. Color code shows the host, the type of sample (train or test the classifier), the initial 16S rRNA gene region amplified, and the geographical origin of the samples. Samples were ordered by source from the most similar samples (left) to the most dissimilar (right) based on the averaged intrasource Bray-Curtis dissimilarity comparisons. h., horse. Bray-Curtis analysis of the V4 *Clostridiales* and V6 *Bacteroidales* and *Clostridiales* assemblages are presented in Fig. S2 and S3 in the supplemental material, respectively.

We focused on *Bacteroidales* and *Clostridiales* to perform fecal source identification, since they are the most dominant fecal microbiota and have the highest genetic diversity among mammal fecal samples that we examined (Fig. 1). Despite microdiversity and geographical patterns within some sources, both taxonomic groups provided resolution to observe source-specific structures in their assemblages (Fig. 2). This structure allowed us to create and store fecal source signatures as classifiers for both bacterial orders and multiple 16S rRNA gene variable regions. A sample was considered contaminated by a source if random forest classification could identify a bacterial structure similar to the fecal signature stored in the classifier.

To create the initial classifiers, we used the 15 to 20 samples with the most similar *Bacteroidales* or *Clostridiales* sequence structure (based on Bray-Curtis intrasource comparisons) for the V4V5 and V6 variable regions. We then tested the specificity (i.e., proportion of correctly classified negative samples) and sensitivity (i.e., proportion of correctly classified positive samples) of these classifiers against samples with the most dissimilar *Bacteroidales* or *Clostridiales* sequence structure. Exceptions were applied when a strong community bifurcation was observed within an animal source assemblage (i.e., for V6 cattle and dog; see Materials and Methods). We maximized the performance of each classifier by tailoring the voting tree probability cutoff (called the decision cutoff) at which a sample was considered contaminated or not by a source (see Materials and Methods); the voting tree probability is a proxy reflecting the bacterial profile similarity between a tested sample and a fecal signature. The performances of classifiers and decision cutoffs are summarized in Table 1. The classifier amplicon sequence variants (ASVs), which are the highly resolved operational taxonomic units composing the individual source signatures, are listed in Table S1 in the supplemental material. All V4V5 and V6 sewage classifiers had a specificity of 100%. All sewage samples were correctly classified. All animal classifiers had a specificity ranging from 82 to 100%, except V4V5 dog *Bacteroidales*, for which no cutoff could be defined; this classifier was therefore discarded. Misclassifications typically involved animals with similar diets, e.g., misclassifications of horse as cattle (see Data Set S1 in the supplemental material for individual sample predictions). The fecal sources with the lowest sensitivity, e.g., dog or cattle, had a large intrasource dispersion, likely due to a larger number of atypical samples used to test the classifier performance (see nonparametric multidimensional scaling plot in Fig. S1 in the supplemental material).

**TABLE 1:** Sensitivity and specificity of the V4V5 and V6 source classifiers

Region	Source	*Bacteroidales*						*Clostridiales*^*a*^					
		Specificity (%)^*b*^	*n*	Sensitivity (%)	*n*	Decision cutoff (%)	Classifier type^*c*^	Specificity (%)^*b*^	*n*	Sensitivity (%)	*n*	Decision cutoff (%)	Classifier type^*c*^
V4V5	Sewage	100	82	100	52	14	Global	100	89	100	53	16	Global
	Dog	82 (cat, raccoon)	125	100	9	14	Global	NA	134	NA	8	ND	Discarded
	Pig	100	123	100	11	14	Global	100	130	100	12	39	Global
V6	Sewage	100	261	100	9	21	Global	100	265	100	9	16	Global
	Dog	91	239	84	31	1	Discarded	87 (cat)	243	100	31	34	Global
	Cattle	82 (horse, kangaroo, raccoon)	222	85	48	26	Global	86 (horse)	226	81	48	26	Global
	Deer	84 (sheep)	257	100	13	4	Discarded	95 (sheep)	261	100	13	26	Global
	Pig	99	235	100	35	14	Global	100	238	100	36	31	Global

a NA, not applicable; ND, not defined.

b Source(s) for which the majority of the samples were misclassified are given in parentheses.

c Type of classifier based on decision cutoff. Classifiers with a decision cutoff of >10% were considered global, and those with a cutoff of ≤5% discarded.

10.1128/mSystems.00869-19.1 DATA SET S1 Sample metadata (separated Excel sheet). Download Data Set S1, XLSX file, 0.1 MB. Copyright © 2020 Roguet et al. 2020 Roguet et al. This content is distributed under the terms of the Creative Commons Attribution 4.0 International license.

10.1128/mSystems.00869-19.2 TABLE S1 List of the sequences composing the V4, V4V5, and V6 classifiers. Download Table S1, XLSX file, 0.1 MB. Copyright © 2020 Roguet et al. 2020 Roguet et al. This content is distributed under the terms of the Creative Commons Attribution 4.0 International license.

10.1128/mSystems.00869-19.7 FIG S1 Nonmetric multidimensional scaling plot indicating the *Bacteroidales* and *Clostridiales* assemblage composition relationships between the sewage and animal fecal samples for the V4 and V6 data sets. Download FIG S1, TIF file, 2.2 MB. Copyright © 2020 Roguet et al. 2020 Roguet et al. This content is distributed under the terms of the Creative Commons Attribution 4.0 International license.

10.1128/mSystems.00869-19.8 FIG S2 Bray-Curtis analysis of the *Clostridiales* assemblage for the V4 region of the 16S rRNA gene. Download FIG S2, TIF file, 2.2 MB. Copyright © 2020 Roguet et al. 2020 Roguet et al. This content is distributed under the terms of the Creative Commons Attribution 4.0 International license.

10.1128/mSystems.00869-19.9 FIG S3 Bray-Curtis analysis of the *Bacteroidales* and *Clostridiales* assemblage for the V6 region of the 16S rRNA gene. Download FIG S3, TIF file, 2.9 MB. Copyright © 2020 Roguet et al. 2020 Roguet et al. This content is distributed under the terms of the Creative Commons Attribution 4.0 International license.

We only considered classifiers to be validated as global classifiers if the specificity and the sensitivity were ≥70% when using a voting tree decision cutoff of >10%. Reducing the voting cutoff further increases sensitivity in some cases but creates specificity tradeoffs and may generate too many misclassifications when testing environmental samples. Classifiers with a cutoff of ≤10% were considered “draft” in order to offer the users the ability to explore their data set while considering uncertainty. Classifiers with a decision cutoff of ≤5% were discarded.

### Scalability of the classifiers.

In addition to the host-specific classifiers, we developed diet/physiology-centered classifiers, including those for herbivore and ruminant sources. These broad classifiers allowed us to extend the scope of the host-specific classifiers to capture fecal signatures shared among hosts having similar diets or physiology. This approach also allowed us to include sources with limited sample sets that prohibited creating individual host classifiers (e.g., horse or sheep). The diet/physiology-centered classifiers had a minimum specificity and sensitivity of 78% (Table 2). Interestingly, in some cases, the broader classifiers were able to identify the fecal signature in samples (e.g., *Bacteroidales* V6 deer samples) where the host-specific classifiers failed (see Data Set S1).

**TABLE 2:** Sensitivity and specificity of the V4V5 and V6 diet and physiology classifiers

Region	Classifier^*a*^	*Bacteroidales*						*Clostridiales*					
		Specificity (%)^*b*^	*n*	Sensitivity (%)	*n*	Decision cutoff (%)	Classifier type^*c*^	Specificity (%)^*b*^	*n*	Sensitivity (%)	*n*	Decision cutoff (%)	Classifier type^*c*^
V4V5	Ruminant	100	121	92	13	13	Global	99	123	95	19	4	Discarded
V6	Herbivore	78 (raccoon, chicken)	162	87	108	40	Global	88 (raccoon)	167	87	107	25	Global

a Classifiers trained using cattle and deer fecal samples.

b Source(s) for which the majority of the samples were misclassified are given in parentheses.

c Type of classifier based on decision cutoff. Classifiers with a decision cutoff of >10% were considered global, and those with a cutoff of ≤5% discarded.

### Importance of sequencing depth.

We evaluated the performance of the source classifiers (*Bacteroidales* and *Clostridiales* for both V4V5 and V6) in seven freshwater river samples for which sewage and cattle fecal contamination had been previously identified (26) using validated quantitative PCR (qPCR) assays (Table 3). Unlike V4V5, V6 classifiers identified the sewage signature in all the samples. The sequencing of the V6 region using the Illumina HiSeq or NextSeq platform provided 4-fold higher depth than the Illumina MiSeq platform used to sequence the V4V5 region. The two V4V5 samples not classified as contaminated with sewage (MKE_18755 and MKE_18765) had the smallest number of sequences matching the classifiers. This increased sequencing depth appeared to provide the information needed to capture the fecal signature in the environmental samples where bacteria are already abundant (in freshwater samples, bacterial abundance is typically >10^5^ cells per ml). For this reason, we decided to focus our classifier approach on short hypervariable regions of the 16S rRNA gene to maximize sequencing depth. In addition to the V6 region, we created and tested the performance of classifiers built on the V4 region, which is targeted extensively in environmental and fecal microbiota data sets (27). We incrementally subsampled all fecal samples used to test the V4 and V6 classifiers in order to define the classifier detection limits. This analysis revealed that a minimum of 1,000 sequences was necessary to properly classify a sample. Additionally, artificial bacterial assemblages composed of several fecal signatures were also correctly classified when the fecal signature matching the classifiers was composed of at least 1,000 sequences represented by ∼100 unique representative sequences (see Table S2 in the supplemental material for individual sample predictions). The relationship between the number of sequences in a sample (proxy of the percentage of contamination) and the voting tree probability was logarithmic.

**TABLE 3:** Random forest classifications using the V4V5 and V6 global classifiers of seven freshwater river samples contaminated by sewage and cattle fecal pollution^*a*^

Sample identifier	Human marker^*b*^	Ruminant marker^*b*^	Total no. of sequences (no. of sequences matching the V4V5 classifiers)	Classifier for:		Total no. of sequences (no. of sequences matching the V6 classifiers)	Classifier for:	
				*Bacteroidales*	*Clostridiales*		*Bacteroidales*	*Clostridiales*
MKE_18755	4.26	4.54	54,495 (25)			212,620 (818)	Sewage	Sewage
MKE_18756	5.71	4.61	51,879 (363)	Sewage	Sewage	296,677 (3,171)	Sewage	Sewage
MKE_18757	5.63	4.66	75,024 (588)	Sewage	Sewage	276,911 (2,585)	Sewage	Sewage
MKE_18759	5.65	5.30	76,611 (732)	Sewage	Sewage	258,374 (2,863)	Sewage	Sewage
MKE_18761	5.63	5.33	72,991 (526)	Sewage	Sewage	345,635 (3,849)	Sewage	Sewage
MKE_18763	5.15	5.78	65,949 (288)	Sewage	Sewage	249,932 (1,442)	Sewage/herbivore	Sewage/herbivore
MKE_18765	5.02	6.22	68,772 (220)	Ruminant	Sewage	332,866 (1,779)	Sewage	Sewage/cattle/deer/herbivore

a V4V5 global classifiers include sewage, dog (*Bacteroidales*), pig, and ruminant (*Bacteroidales*). V6 global classifiers include sewage, dog (*Clostridiales*), cattle, pig, deer (*Clostridiales*), and herbivore.

b Copy number per 100 ml (log_10_-transformed). Human and ruminant quantitative PCR marker data from Olds et al. (26).

10.1128/mSystems.00869-19.3 TABLE S2 *In silico* artificial community predictions generated using the V4 and V6 classifiers. Download Table S2, XLSX file, 0.01 MB. Copyright © 2020 Roguet et al. 2020 Roguet et al. This content is distributed under the terms of the Creative Commons Attribution 4.0 International license.

### Flexibility of sequence data.

We developed and compared V4 classifiers from both *de novo*-produced V4 sequence data and trimmed V4V5 sequence reads. The ability to reliably use trimmed data was explored using 42 untreated sewage and 191 animal fecal samples sequenced in both the V4 and V4V5 regions. The V4 primer sites were recognized in more than 99% of *Bacteroidales* and *Clostridiales* V4V5 reads, suggesting that the V4 primer sites are highly conserved in these two bacterial orders. Since the trimmed V4V5 data set was sequenced to a lower depth, we rarefied the *de novo* V4 data set to the median count of trimmed V4V5 reads. We created three source classifiers, namely sewage, cattle, and pig, using both rarefied *de novo* V4 and trimmed V4V5 data sets. The majority of the sequences composing the classifiers were shared between the two data sets (see Table S3 in the supplemental material). Moreover, they generated comparable sensitivity and specificity metrics when tested using their respective data set and the reciprocal data set (i.e., rarified *de novo* V4 classifiers tested using trimmed samples and *vice versa*). Interestingly, a nonrarefied *de novo* V4 classifier had lower sensitivity when using trimmed V4V5 data, likely because of the lower sequence depth of the test data set. The performance of each configuration is described in Table S4 in the supplemental material. Thereafter, V4 classifiers exploiting both rarefied *de novo* V4 and trimmed V4V5 data sets were implemented in FORENSIC. The performance is described in Table 4.

**TABLE 4:** Sensitivity and specificity of the combined V4 classifiers generated using *de novo* V4 and trimmed V4V5 sequences to the V4 primer positions

Classifier	*Bacteroidales*						*Clostridiales*					
	Specificity (%)^*a*^	*n*	Sensitivity (%)	*n*	Decision cutoff (%)	Classifier type (%)^*b*^	Specificity (%)^*a*^	*n*	Sensitivity (%)	*n*	Decision cutoff (%)	Classifier type^*b*^
Sewage	100	138	100	56	19	Global	100	142	100	57	17	Global
Dog	87 (cat)	185	100	9	22	Global	75 (cat, raccoon)	190	78	9	10	Draft
Cattle	100	161	100	33	9	Draft	99	162	100	37	16	Global
Pig	99	168	100	26	13	Global	99	174	100	25	29	Global
Ruminant^*c*^	93 (kangaroo)	150	100	44	5	Discarded	97 (kangaroo)	151	100	48	10	Draft
Herbivore^*d*^	88 (raccoon, goose)	135	95	59	33	Global	99	136	89	63	22	Global

a Source(s) for which the majority of the samples were misclassified are given in parentheses.

b Type of classifier based on their decision cutoff. Classifiers with a decision cutoff of >10% were considered global, those with a decision cutoff of >5% were considered draft, and those with a cutoff of ≤5% were discarded.

c Ruminant classifiers trained using cattle and deer fecal samples.

d Herbivore classifiers trained using cattle, deer, and horse fecal samples.

10.1128/mSystems.00869-19.4 TABLE S3 List of the sequences composing the rarefied *de novo* V4 and trimmed V4V5 classifiers. Download Table S3, XLSX file, 0.05 MB. Copyright © 2020 Roguet et al. 2020 Roguet et al. This content is distributed under the terms of the Creative Commons Attribution 4.0 International license.

10.1128/mSystems.00869-19.5 TABLE S4 Comparison of the performance of the sewage, dog, and pig classifiers generated using *de novo* V4, rarefied *de novo* V4, and trimmed V4V5 data sets and tested against *de novo* V4 and trimmed V4V5 samples. Download Table S4, XLSX file, 0.01 MB. Copyright © 2020 Roguet et al. 2020 Roguet et al. This content is distributed under the terms of the Creative Commons Attribution 4.0 International license.

### FORENSIC implementation and user interface.

FORENSIC integrates the validated classifiers to perform fecal source identification and can be accessed online at https://forensic.sfs.uwm.edu/. It requires a FASTA file (see Materials and Methods) and yields source contamination predictions in raw downloadable text and interactive visualizations. Classification relies upon an exact match with fecal signature sequences, and therefore trimming to exact V4 or V6 primer positions is crucial. Most FORENSIC analysis takes a few seconds to a few minutes, depending on the number of input sequences. FORENSIC currently encompasses a total of five global source classifiers and two physiology/diet classifiers for V4 and/or V6 regions (Tables 1, 2, and 4).

**FIG 3:**
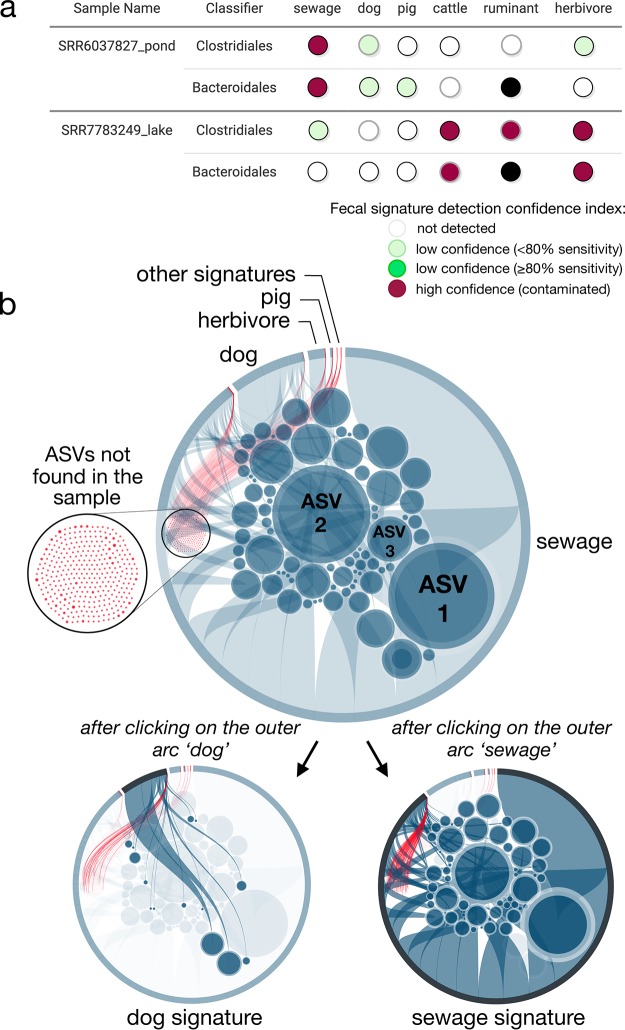
Forensic interactive report, including (a) a source identification predictions table and (b) bubble plot to characterize the fecal signature in the submitted samples. (a) Colors indicate if the fecal source signature was characterized (red) or not (white or green) in the tested sample. Dark green sources indicate that at the voting tree probability observed, the specificity of the classifier was at least 80%; light green sources indicate a specificity of <80%. Global classifiers are represented by black rings. Discarded classifiers are symbolized using black circles. (b) Bubbles represent all the amplicon sequence variants (ASVs) that compose the classifiers for a given bacterial group. Blue bubbles show the ASVs recovered from the sample tested, unlike red bubbles. The sizes of the bubbles are proportional to the relative abundances of the ASV in the tested sample. Sources are represented by the outer arcs. The longer the arc is, the more that source contributed to the fecal pollution (among the sources investigated). The example shows the V4 *Clostridiales* profile recovered from a Chinese surface-water pond (SRA accession number SRR6037827) and classified as sewage by random forest. A total of 74 out of the 385 ASVs that compose all the classifiers were found in the sample; 55 and 14 ASVs were associated with the sewage and dog classifiers, respectively. The largest arc (representing 91% of the relative abundance of all the fecally associated ASVs) was associated with the sewage signature (bottom right). The second largest was associated with the dog and the third with the pig signature. The interactive version of this figure is available at https://forensic.sfs.uwm.edu/result/example. Data from Hägglund et al. and Hu et al. (16, 63). See Table S5 in the supplemental material for the full list of the individual predictions.

FORENSIC visualizes its predictions in multiple ways, including a color-coded table of source category predictions for each submitted sample (Fig. 3a) and an interactive association networks of ASVs and sources that allows the user to explore and disentangle the different fecal profiles recovered from the samples (Fig. 3b). This interactive visualization strategy can also help to detect potentially false-positive predictions by discerning the prevailing fecal signature(s) from other trace source signatures and/or from signatures sharing sequences with the dominant one. Users can access raw data outputs such as counts of sequences matching to each classifier, voting tree probabilities, and simplified identifications. Warning flags are displayed if samples do not meet certain criteria, such as the number of sequences in the submitted FASTA or the number of sequences (or unique representative sequences) matching to the classifiers being too low indicating insufficient sequencing depth or, in the case of no matching sequences, technical issues in data processing.

10.1128/mSystems.00869-19.6 TABLE S5 Fecal predictions using the global classifiers of the sewage, animal fecal, and environmental samples from Hägglund et al. (16) and Hu et al. (63). Download Table S5, XLSX file, 0.03 MB. Copyright © 2020 Roguet et al. 2020 Roguet et al. This content is distributed under the terms of the Creative Commons Attribution 4.0 International license.

## DISCUSSION

FORENSIC is an online platform for performing fecal source identification in water from 16S rRNA gene amplicons. It leverages *Bacteroidales* and *Clostridiales*, two bacterial orders with many members that show remarkable host specificity in mammalian guts (15, 23, 28, 29). Microbial community differences that distinguish animals are substantially diet driven due to the specialization of bacterial members for specific metabolic functions (30–32). Here, we used these host-microbiota association patterns to discriminate fecal contamination with both host-based (e.g., human, cattle, pig) and/or diet/physiology-based (e.g., ruminant, herbivore) classifiers. Specific markers for both Bacteroides (33, 34) and Lachnospiraceae (35) have shown a broad geographic distribution. Consistent with these findings, we observed minimal geographic sample partitioning for the whole *Bacteroidales* and *Clostridiales* assemblages, which demonstrates that these classifiers could have global relevance.

The FORENSIC sewage classifiers had a sensitivity of 100%, meaning that all tested sewage samples were correctly classified. These samples were collected from three continents, which confirms that sewage carries a robust human fecal signature (36, 37) and that the classifiers are potentially applicable to cities worldwide with modern sewer infrastructure. The sewage signature also is unique and differentiated from the other nonintegrative (individual animal) sources, as no animal fecal samples were identified as sewage and no sewage samples were classified as an animal source.

Given that the microbial community composition of a single fecal sample can be quite distinct from that of an integrative sample (36), we wanted to test whether this approach could correctly identify sources from samples encompassing the variability expected among individual sources. By creating classifiers using only the 15 to 20 most similar samples and then testing their performance using the most dissimilar samples, we were able to examine this scenario. The random forest approach generated high sensitivity and specificity for most fecal pollution sources we tested, demonstrating the consistency and cohesion of the host fecal signature as characterized by a minimum number of samples. Misclassifications generally involved sources with similar diets or samples with atypical fecal microbiota. Given that fecal pollution in waterways is generally derived from multiple animals, we anticipate that the application of FORENSIC for actual water sample testing would produce few false-negative results and that sporadic individual atypical animals would not influence the results.

For two animal sources (cattle and dog), the community composition data split into two very distinct community types, possibly driven by the diet of the animal or other factors such as cohabitation, age, or individual variation. For instance, a large dissimilarity in the cattle microbial community composition has been observed between cattle that are grass fed versus those that are grain fed (38, 39). Therefore, specific animal groups, such as cattle, that are fed distinct diets or have other distinct lifestyle characteristics may not be suitable for a single-marker-gene or classifier approach for tracking their impact. Instead, multiple classifiers used in combination may be needed to cover the diversity of a single animal group.

Global classifiers implemented in FORENSIC were characterized from a minimum of 15 source samples and had a specificity and sensitivity of 70% and a decision cutoff of >10%. Classifiers that did not meet these requirements were either discarded or designated draft classifiers. Cats and dogs had microbial communities similar to that of sewage, indicating shared sequences (Fig. S1). This may be due to shared diet or to actual pet or wildlife inputs into the sewer conveyance system. We did not attempt to make a cat classifier, and two of the four dog classifiers were either discarded or designated draft.

FORENSIC is implemented with random forest classification, which is particularly suitable for an online application, especially since the classifiers do not require each new investigation to reanalyze *de novo* bacterial assemblages in all trained and tested samples. The global classifiers of FORENSIC are based on the shared/common source signatures, increasing access to sequence-based analysis for users who do not have source samples. SourceTracker (21), a well-established tool, relies upon direct comparison of a source community to the sink community and requires both communities to be used as inputs. Users would need to provide representative samples, or chose samples from publicly available data. Analysis is performed *de novo*, i.e., without a reference database of what to expect, and therefore does not require that a source contain a universal signature. SourceTracker is highly sensitive, particularly for atypical sources or uncharacterized sources, when the source community is available. Furthermore, since the entire community is used for comparison, SourceTracker may be more suitable to identify animals where additional taxonomic groups provide a more distinctive signature than *Bacteroidales* or *Clostridiales*. Closely related animals (cattle and horses), unique geographic signatures, or the presence of uncharacterized sources, particularly wildlife, should be considered when choosing a tool.

The scalability of FORENSIC makes it possible to add more samples to the reference database as they become available. This characteristic will allow for systematic verification of the classifier performance, with a goal of continuous improvement. Overall, future directions include (i) extending the source classifiers to include farm animals or waterfowl and targeting predominant bacterial groups appropriate to these sources, (ii) strengthening broad diet/physiology classifiers, or, inversely, (iii) characterizing finer subsource clusters to improve the sensitivity of the classifiers.

Our study also highlights that sequencing depth is crucial to capturing fecal signatures within environmental samples. Even at relatively significant contamination levels, the fecal microorganisms can be outnumbered by the native microorganisms by >2 orders of magnitude. We recommend implementing our approach on deeply sequenced data (i.e., >100,000 reads) and using sequencing platforms that generate shorter reads (e.g., V4 or V6 regions of the 16S rRNA gene) and therefore greater depth. However, FORENSIC correctly identified the source of fecal pollution in environmental samples with lower sequencing depth where the fecal signature was represented with a minimum of 1,000 sequences and 100 unique representative sequences. This rate of correct identification equates to correctly identifying the fecal signature in 100,000 reads if the signature comprises 1% of the community.

When generating the classifier, the short V4 reads performed as well as the longer V4V5 reads, which supports previous reports that minimal information was needed for resolution of microbial members (40, 41). Although primers for the region targeted are often cited for introducing bias in the assessment of microbial communities (42, 43), we demonstrated the equivalence of using V4V5 sequences trimmed to the Earth Microbiome Project V4 primer positions and of using *de novo* V4 sequences for the specific FORENSIC application. This result is congruent with previous studies that reported a limited bias for several phyla, including Bacteroidetes and Firmicutes (44, 45), likely because primer sites are conserved within closely related groups.

In summary, FORENSIC offers a comprehensive open-source platform that enables rapid and straightforward screening of large sequence data sets to identify fecal contamination. In the era of affordable and accessible high-throughput sequencing, FORENSIC is a valuable tool for community-based water quality assessments to prospect the main sources of fecal pollution and guide appropriate management actions.

## MATERIALS AND METHODS

### Sample collection and processing.

Animal stool samples were collected in the United States, France, the Dominican Republic, and Australia. Samples collected in the United States were preserved in lysis buffer from the stool extraction kit and shipped to the laboratory on ice and stored at −80°C upon arrival. Dominican Republic samples were stored at −20°C until extracted. Australian fecal sample processing and storage were described previously, and extracted DNA was provided (46). Animal stool samples from France were freeze-dried prior to DNA extraction. A human fecal signature was characterized from sewage influent samples from 71 cities across the United States (36), Reus in Spain (47), and Sydney in Australia. Finally, seven river samples collected after heavy rainfall in the Milwaukee River Basin, Milwaukee, WI, USA, were used to test the sensitivity of the classifiers (26). Sewage and animal fecal sample details are reported in Data Set S1.

### Sample processing and DNA extraction.

For the U.S. and Dominican Republic fecal samples, bacterial DNA was extracted from approximately 1 g of material using a QIAmp DNA stool minikit according to the manufacturer’s instructions (Qiagen, Valencia, CA) as described (25). About 200 mg of Australian stool samples was extracted using the QIAmp stool DNA kit, while about 500 mg of dry fecal sample for French samples was extracted using the FastDNA spin kit for soil (MP Biomedicals, Carlsbad, CA). Australian and French extracted DNA was freeze-dried before being shipped to the United States.

In total, 25 ml for sewage influent and between 200 and 400 ml for freshwater samples was filtered onto 0.22-μm mixed cellulose ester filters with a 47-mm diameter (Millipore, USA). DNA from filters was then extracted using the FastDNA spin kit for soil (MP Biomedicals, Solon, OH) as previously described (26, 36). DNA concentration was assessed on a Qubit 2.0 fluorometer (Invitrogen, Carlsbad, CA).

### 16S rRNA gene sequencing and library construction.

Amplicon libraries were constructed at the Josephine Bay Paul Center in the Marine Biological Laboratory (Woods Hole, MA) and/or the Great Lake Genomics Center (Milwaukee, WI) using the MiSeq Illumina platform for the V4 and V4V5 hypervariable regions and the HiSeq and/or NextSeq Illumina platforms for V6 hypervariable regions. Details for amplicon library construction and sequencing procedures for the V4V5 and V6 regions were followed as previously described (48, 49). The construction of the V4 amplicon library using the Earth Microbiome primers is described in Text S1 in the supplemental material.

10.1128/mSystems.00869-19.10 TEXT S1 V4 amplification and sequencing. Download Text S1, DOCX file, 0.02 MB. Copyright © 2020 Roguet et al. 2020 Roguet et al. This content is distributed under the terms of the Creative Commons Attribution 4.0 International license.

Reads were trimmed using cutadapt v1.14 (50), allowing for two to four mismatches in the primer sequence depending on the region. Forward and reverse reads were merged using PEAR v0.9.10 (51) with the default parameters. Merged reads with a length shorter or longer than 5% of the V4V5 median (372 bp) or 10% of the V4 and V6 median (i.e., 253 and 60 bp, respectively) were removed during this step. Quality filtering was assessed using mothur 1.39.5 (52). Assembled reads containing ambiguous bases or with more than eight successive homopolymers were discarded. Sequences were taxonomically assigned based on the best match in a Global Alignment for Sequence Taxonomy (GAST) (53) process using the Silva 132 database (54). Only sequences assigned to *Bacteroidales* and *Clostridiales* were selected for further analysis.

### Minimum entropy decomposition analysis.

Minimum entropy decomposition (MED) analysis was performed using the oligotyping pipeline version 2.1 (55). MED uses nucleotide entropy (nucleotide variant variability) along DNA sequences to distinguish differences in nucleotides originating as a result of true genetic variation among organisms from noise due to sequencing errors (56). MED partitions DNA sequences into amplicon sequence variants (ASVs) according to the position in the DNA sequence with the highest entropy. This step iteratively lasts until each final ASV satisfies the maximum entropy criterion. ASVs that do not meet the minimum substantive abundance (M) criterion were discarded. M was set to N/30,000, N/40,000, and N/60,000, for the V4V5, V4, and V6 data sets, respectively, where N was the total number of sequences in the data set.

### Selection of the samples to train and test the classifiers.

Sequencing quality was systematically evaluated for each sewage and animal fecal sample before creating or testing the classifiers. For that, a prospective MED analysis was performed for both *Bacteroidales* and *Clostridiales* using all the samples of a given source. Samples in which more than 50% of the reads were discarded during the MED analysis for at least one of the bacterial groups were deleted from further analysis. The total numbers of amplicon sequence variants (ASVs) were log_10_ transformed and plotted in a box plot. Lower outlier samples, i.e., those lower than 1.5 times the interquartile, were considered to have low sequencing depth and were discarded. We also verified for each source that there was no sample with an extremely atypical bacterial assemblage using Bray-Curtis dissimilarities computed separately for each source and bacterial group via the *vegan* package (57) implemented in R. Intrasource dissimilarities were averaged per sample and plotted in a box plot; upper outliers were not used to evaluate the performance of the classifier but are reported separately in Data Set S1. These extreme outliers comprised less than 8% of the total samples and, in some cases, had Bray-Curtis dissimilarity scores of >0.95 compared to other samples from a given host, which could have resulted from sample handling, external contamination, or poor sequencing quality.

Each classifier was built using 15 to 20 samples having the most similar intrasource bacterial assemblages (based on Bray-Curtis dissimilarity index). The validation of the classifier predictions was assessed with a minimum of six samples (30% minimum of the samples per source) having the most dissimilar intrasource bacterial assemblages to ensure a stringent assessment of performance. We systematically explored the intrasource variability of the bacterial group assemblages using a hierarchical clustering analysis (based on the Bray-Curtis dissimilarity and Ward linkage) in order to take into account environmental factors that may result in bifurcation of gut microbiota structure within sources. We defined subgroups within sources when the branch length between two clusters for a given source was greater than five. A relevant shift was observed in the V6 cattle (*Bacteroidales* and *Clostridiales*) and V6 dog (*Bacteroidales*) bacterial assemblages. A balanced number of samples from each cluster was used to build the V6 cattle and dog (*Bacteroidales*) classifiers. Diet and physiology classifiers were trained and tested using the collection of samples for each respective category (e.g., ruminant trained and tested using cattle and deer samples).

### Random forest classifications.

To characterize the fecal source signature for a classifier, bacterial assemblages from a given source (e.g., sewage) were compared to the assemblages from all other source samples (e.g., nonsewage samples from dog, cattle, etc.). For that, 100 random forests consisting of 10,000 trees were computed per source and per bacterial group using the default settings of the ‘randomForest’ function implemented in the *randomForest* R package (58). Mean decreases in Gini were averaged for each ASV among the 100 random forest replicates. Mean decreases in Gini were plotted in a scree plot. ASVs with mean decreases in Gini above the breakpoint curve were chosen to be part of the classifier. Breakpoints were estimated using the “breakpoints” function included in the *strucchange* R package (59). Classifiers were trained with 100 forests constituted of 1,000 trees to recognize the fecal source signatures from the ASVs selected upstream (per source and bacterial group, the total relative abundance of the ASVs was of 100%). Replicates were pooled using the “combine” function. One classifier was created for each source, bacterial group, and region of the 16S rRNA gene. To identify the fecal source signature in a test sample, sequences matching the ones in the classifiers were first extracted. Bacterial profiles were then compared to the ones stored in the classifiers using the “predict” function, which generated a voting tree probability, translating the degree of similarity between the test and the classifier profiles. We defined the voting tree probability cutoff (called the decision cutoff) to maximize the sensitivity and specificity with a minimum of 70% for each classifier. Samples associated with a voting tree higher than the decision cutoff for a given source were considered to be contaminated by that source. In addition to the source-specific classifiers (e.g., sewage, cattle), we created diet/physiology classifiers from multiple fecal source samples: V4/V4V5 ruminant classifiers were created using cattle and deer fecal samples, and a V4 herbivore classifier was created using cattle, deer, and horse fecal samples. Although V6 herbivore classifiers were created using ruminant fecal samples (i.e., cattle and deer), their specificity to only detect the ruminant fecal signature was low compared to that for detecting the herbivore signature (data not shown). Thus, these classifiers were considered herbivore classifiers. All analyses were conducted using the statistical environment R (version 3.6.0) (60).

### Sensitivity, specificity, and library size detection limit of random forest classifications.

Sensitivity was calculated as the number of true positives (TPs) (i.e., when a source was correctly detected) divided by the sum of TPs and false negatives (FNs) (i.e., when a source was not detected as expected). In contrast, specificity was defined as the number of true negatives (TNs) (i.e., when a source did not have a classifier and thus should have been classified as “unknown”) divided by the sum of TNs and false positives (FPs) (i.e., when an unexpected source was detected). Classifier performance for specificity was evaluated using the test samples from host groups that were the focus of the classifier, but with also seven additional animal fecal sources; see Data Set S1 for details. The minimum number of sequences at which a classifier can correctly classify a fecal sample was assessed using an *in silico* analysis. All V4 and V6 test samples were subsampled at 90, 80, 70, 60, 50, 40, 30, 20, 10, 5, 1, 0.5, 0.1, 0.05, 0.01, 0.005, and 0.001%. The subsampling was performed using the function “sub.sample” in mothur 1.39.5. Additionally, the minimum number of sequences belonging to the classifier signatures at which a classifier can correctly identify multiple fecal sources was assessed using artificial bacterial assemblages generated *in silico*. Defined sequence proportions from pristine surface freshwater and fecal samples (i.e., sewage, cattle, pig, and sheep) not used to train the classifiers were combined to create V4 and V6 artificial bacterial community mixes (see Table S2 in the supplemental material). Sequences from each sample were selected by randomly subsampling (99 repeats) using the “rrarefy” function included in the *vegan* R package.

### Implementation of the online interface.

The FORENSIC Web interface (https://forensic.sfs.uwm.edu/) was designed using free and open software development practices in mind. The source code of FORENSIC was implemented in Python programming language using the Flask Web Framework (https://palletsprojects.com/p/flask/) for easy maintenance. FORENSIC can run as a Web application behind a reverse proxy (such as NGinx) and on Web server (such as Apache; http://apache.org/), and can be deployed on any Unix-based operating system. The reverse proxy handles Hypertext Transfer Protocol Secure (HTTPS) encryption for security, sanitization of malformed inputs, and efficient data upload. FORENSIC requires the upload of a multi-FASTA file and assumes the sequencing reads to have been trimmed and merged. The Python Web application detects the 16S rRNA region targeted in sequences and generates a count table of the ASVs matching the sequences in the classifiers. After the classification, the Web platform generates a static report using the data generated. The interactive report page visualizations are created using D3.js (https://d3js.org/) and our *ad hoc* JavaScript code. The data used for visualizations can also be directly downloaded from the interface as tab-separated data files for user-directed analyses. The web platform also notifies the user via e-mail when analysis is complete if an e-mail address has been provided during submission. A unique identifier (ID) is also provided during the submission to review the results for up to 1 year.

### Data availability.

The source code of FORENSIC is available online (https://github.com/mclellanlab/web_forensic) and licensed with the General Public License to allow local and institutional deployments. The R scripts to create, train, and test the classifiers are available on figshare, as well as the script to determine the decision cutoff (https://doi.org/10.6084/m9.figshare.11231627). Raw read files were uploaded to the National Center for Biotechnology Information (NCBI) Sequence Read Archive (SRA) and are accessible under the BioProject accession numbers PRJNA261344, PRJNA264400, PRJNA433408, PRJNA591968, and PRJNA591970 for V4V5; PRJNA591976 and PRJNA591975 for V4; and PRJNA235337, PRJNA433407, PRJNA591949, and PRJNA505345 for V6. Data Set S1 contains the list of samples and their corresponding SRA study accession numbers.
