# Supplementary figures and images for: FORENSIC: an Online Platform for Fecal Source Identification
Source: mSystems. 2020 Mar 17;5(2):e00869-19. doi: 10.1128/mSystems.00869-19 (PMC7380585; doi:10.1128/mSystems.00869-19)

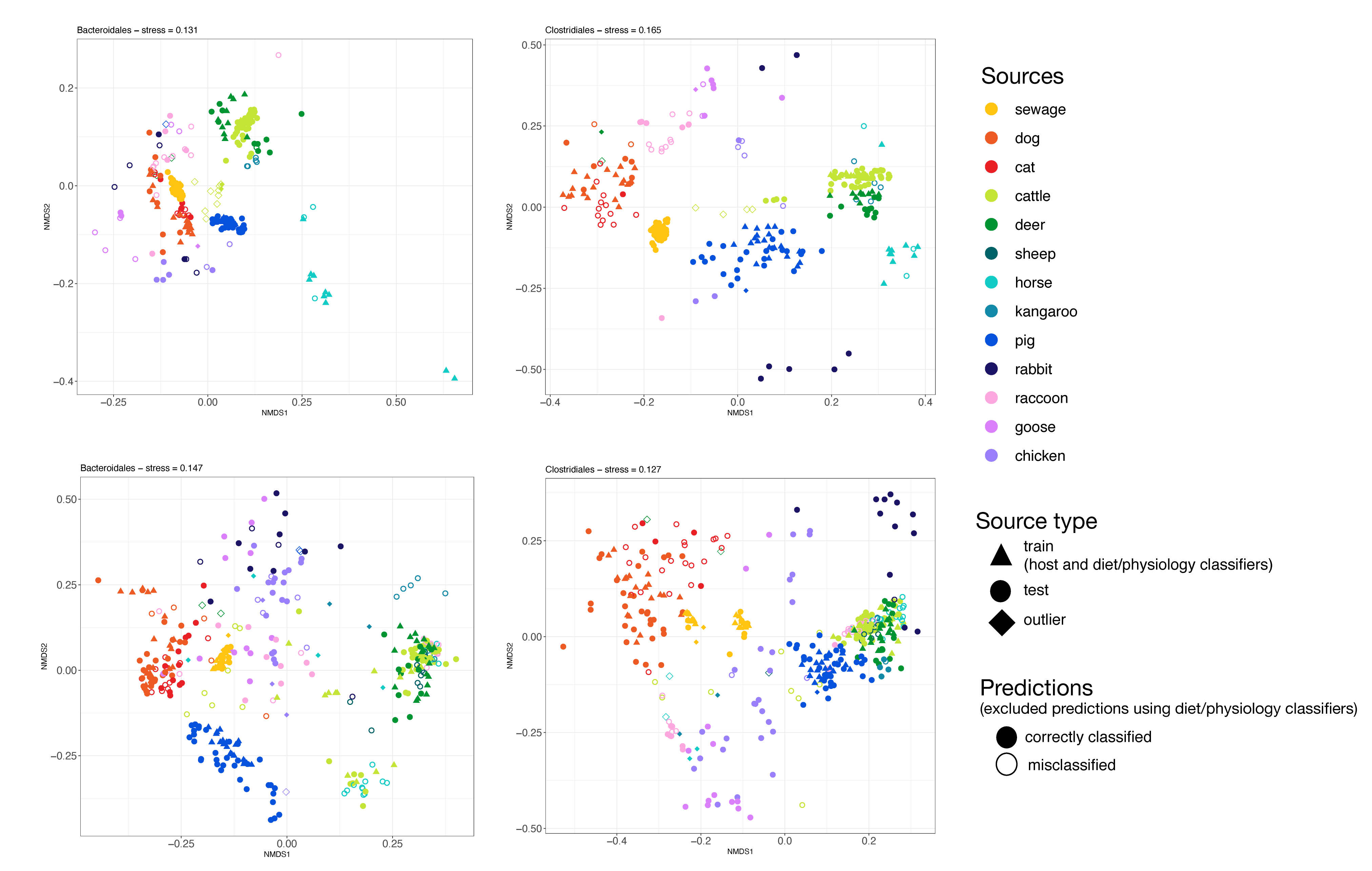

Supplement: FIG S1 [file mSystems.00869-19-sf001.tif]

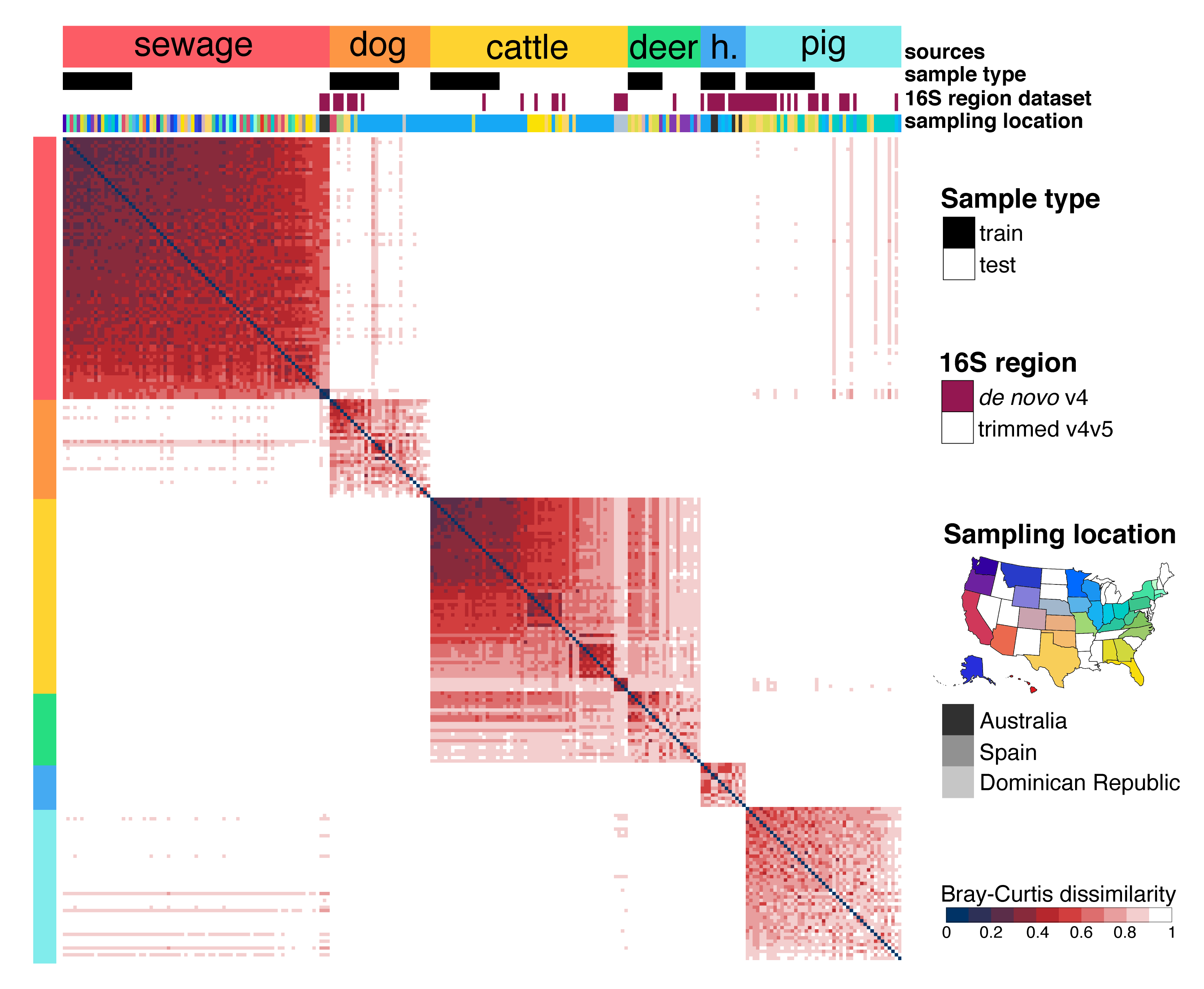

Supplement: FIG S2 [file mSystems.00869-19-sf002.tif]

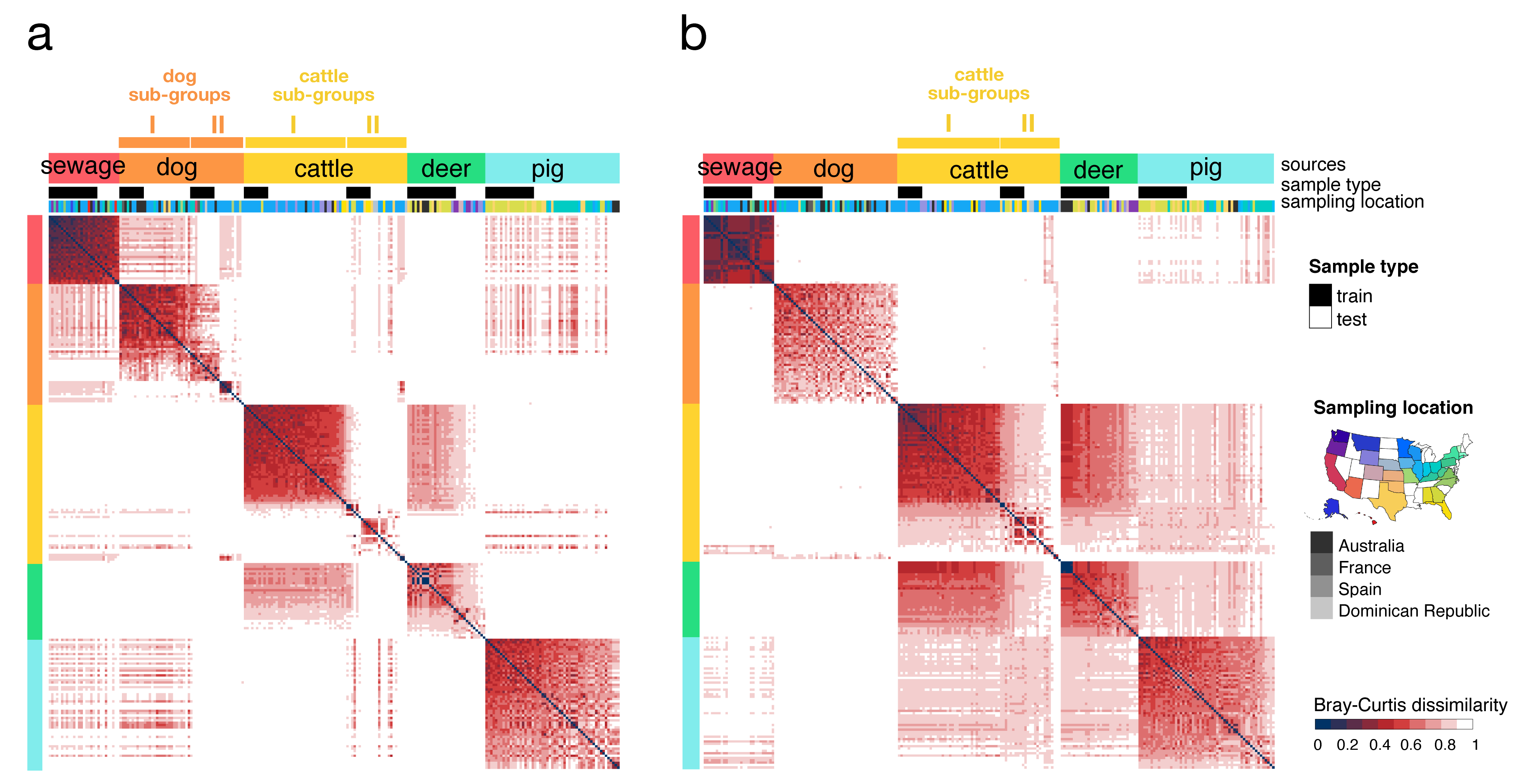

Supplement: FIG S3 [file mSystems.00869-19-sf003.tif]
