# Supplementary material for: FORENSIC: an Online Platform for Fecal Source Identification
Source: mSystems. 2020 Mar 17;5(2):e00869-19. doi: 10.1128/mSystems.00869-19 (PMC7380585; doi:10.1128/mSystems.00869-19)
Supplement: TEXT S1 [file mSystems.00869-19-s0001.docx]

**Supplementary Methods:** **V4 amplification and sequencing**

### Earth Microbiome Project universal prokaryote primers 515F-Y (Parada et al. 2016; 5’-GT-GTGYCAGCMGCCGCGGTAA-3’) and 806RB (Apprill et al., 2015; 5’-CC-GGACTACNVGGGTWTCTAAT-3’) targeting the V4 region of the 16S SSU rRNA were used to generate the amplicon libraries. The first two bases of each primer are a 'pad' selected so that the longer primer does not match known sequences to avoid preferential amplification. Unlike the protocol described in Caporaso et al. (2012), in which custom sequencing primers were designed to be complementary to the V4 amplification primers to avoid primer sequencing, we used standard Illumina sequencing primers to capture the in-line barcode and primer as part of the quality control process.

Custom fusion primers for PCR consisted of the Illumina adaptor, one of 8 different inline barcodes (forward primer) or one of 12 dedicated indices (reverse primer), and the V4 primer sequence. This use of 96 unique barcode-index combinations allows multiplexing 96 samples per lane. Primers were synthesized by Integrated DNA Technologies.

We carried out the PCR in triplicate 33 uL reaction volumes with an amplification cocktail containing 1.3 U Platinum SuperFi Polymerase (ThermoFisher Scientific, cat. no. 12351050), 1X SuperFi buffer, 200 uM dNTP mix (ThermoFisher Scientific, cat. no. R0193), and 0.16 uM of each fusion primer. We added approximately 10–25 ng template DNA to each PCR and ran a no-template control for each primer pair. Products were amplified for 30 cycles of 94^o^C (45 seconds), 50^o^C (60 seconds), 72^o^C (90 seconds) with an initial denaturation at 94^o^C for 3 minutes and a final extension at 72^o^C for 10 minutes.

The triplicate reaction volumes were pooled after amplification and purified using a 0.75:1 ratio of AMPure XP beads (BeckmanCoulter) to PCR product. Purified DNA was eluted in 20 uL of nuclease-free H_2_O. PicoGreen quantitation (Life Technologies, Carlsbad CA) provided a basis for pooling equimolar amounts of product. After size-selecting pooled products of ~500 bp using Pippin Prep (SageScience, Beverly MA), we employed qPCR (Kapa Biosystems, Woburn MA) to measure concentrations prior to sequencing on the Illumina MiSeq using a 2 x 300 bp format (MiSeq v3). PhiX DNA was spiked in at ~10% and served as a run quality control. The combination of MiSeq Control Software (v3.0.0) to identify reads by index and a custom python script that resolved barcodes enabled complete demultiplexing.

**References**

Apprill A, McNally S, Parsons R, Weber L. 2015. Minor revision to V4 region SSU rRNA 806R gene primer greatly increases detection of SAR11 bacterioplankton. Aquatic Microbial Ecology, 75:129–137. <http://doi.org/10.3354/ame01753>

Caporaso JG, Lauber CL, Walters WA, Berg-Lyons D, Huntley J, Fierer N, Owens SM, Betley J, Fraser L, Bauer M, Gormley N, Gilbert JA, Smith G, Knight R. 2012. Ultra-high-throughput microbial community analysis on the Illumina HiSeq and MiSeq platforms. ISME J 6:1621–1624. <http://doi.org/10.1038/ismej.2012.8>

Parada AE, Needham DM, Fuhrman JA. 2016. Every base matters: assessing small subunit rRNA primers for marine microbiomes with mock communities, time series and global field samples. Environ Microbiol 18:1403–1414. <http://doi.org/10.1111/1462-2920.13023>
